# Heterologous Ad26.COV2.S Prime and mRNA-Based Boost COVID-19 Vaccination Regimens: The SWITCH Trial Protocol

**DOI:** 10.3389/fimmu.2021.753319

**Published:** 2021-09-24

**Authors:** Roos S. G. Sablerolles, Abraham Goorhuis, Corine H. GeurtsvanKessel, Rory D. de Vries, Anke L. W. Huckriede, Marion P. G. Koopmans, Melvin Lafeber, Douwe F. Postma, Debbie van Baarle, Leo G. Visser, Virgil A. S. H. Dalm, Neeltje A. Kootstra, Wim J. R. Rietdijk, P. Hugo M. van der Kuy

**Affiliations:** ^1^ Department of Internal Medicine, Erasmus Medical Center, Rotterdam, Netherlands; ^2^ Department of Hospital Pharmacy, Erasmus University Medical Center, Rotterdam, Netherlands; ^3^ Center of Tropical Medicine and Travel Medicine, Department of Infectious Diseases, Amsterdam University Medical Centers, Amsterdam, Netherlands; ^4^ Infection & Immunity, Amsterdam Public Health, University of Amsterdam, Amsterdam, Netherlands; ^5^ Department of Viroscience, Erasmus Medical Center, Rotterdam, Netherlands; ^6^ Department of Medical Microbiology and Infection Prevention, University Medical Center Groningen, University of Groningen, Groningen, Netherlands; ^7^ Department of Internal Medicine, University Medical Center Groningen, Groningen, Netherlands; ^8^ Center for Infectious Disease Control, National Institute for Public Health and the Environment, Bilthoven, Netherlands; ^9^ Department of Infectious Diseases, Leiden University Medical Center, Leiden, Netherlands; ^10^ Department of Internal Medicine, Division of Allergy & Clinical Immunology and Department of Immunology, Erasmus University Medical Center, Rotterdam, Netherlands; ^11^ Department of Medical Microbiology, Amsterdam University Medical Centers, Amsterdam Infection and Immunity Institute, University of Amsterdam, Amsterdam, Netherlands

**Keywords:** SARS-CoV-2, COVID-19, Homologous vaccination regimen, Heterologous vaccine regimen, randomized clinical trial, Ad26.Cov2.S, BNT162.b2, mRNA1273

Currently, four COVID-19 vaccines are authorized for use in the European Union by the European Medicines Agency (EMA), i.e., Ad26.COV2.S (1), ChAdOx1 nCoV-19 (2), mRNA1273 (3), and BNT162b2 (4). A boost (second vaccination) after the prime (first vaccination) is given for the latter three, to induce a strong and durable immune response (5). The prime and boost vaccines are administered in a so-called “homologous” vaccination regimen, meaning that both given vaccinations are identical.

Despite the unprecedented speed of vaccine development, vaccination programs have been impacted by unforeseen delays in production, logistic challenges, and the need for adaptation of vaccination schedules in response to emergence of more transmissible variants. Combining different vaccines in “heterologous” vaccination regimens would enable more flexible vaccination programs in the future, which would facilitate fast-track implementation and reduce the impact of any supply-chain disruptions (5). Several studies in mice have shown that combining different vaccines (two-dose heterologous vaccination strategy) elicits a broader immune response (neutralizing antibodies and T-cell responses) (6, 7). This is in line with initial COVID-19 trial results in humans, which found that a heterologous prime-boost induces a broader immune response (8, 9).

From an immunological point of view, heterologous vaccination regimens could provide a broader and more robust immune response. Several studies in humans investigate the reactogenicity and immunogenicity of such regimens, combining ChAdOx1 nCoV-19 with BNT162b2 vaccines, in Great Britain [Com-COV 1 and 2 (10)], Germany (11), and Spain [CombiVacS (12)]. The Com-COV 1 study showed that heterologous regimens with a 4-week prime-boost interval, combining ChAdOx1 nCoV-19 and BNT162b2 vaccines, induced superior immune responses compared to homologous regimens. The study reported significantly higher neutralizing antibody titers and higher T cell responses. The results of the Com-COV2 study, combining ChAdOx1 nCoV-19 with either mRNA1273 or NVX-CoV2373 are yet to be published (13). In addition, a heterologous ChAdOx1 nCoV-19/BNT162b2 immunization regimen with a 10-12 week vaccine interval was shown to be as least as immunogenic compared to homologous BNT162b2 vaccination with a three week prime-boost interval (11). The CombiVacS (12) study shows that a second dose of BNT162b2 administered 8-12 weeks after a prime with ChAdOx1 nCoV-19 elicited a robust immune response. As for reactogenicity in heterologous regimens, an increase in reactogenicity after the booster dose was observed in the ChAdOx1 nCoV-19/BNT162b2 immunization regimen (14). In contrast, a prospective study reported comparable reactogenicity between heterologous (ChAdOx1 nCoV-19/BNT162b2) and homologous (BNT162b2/BNT162b2) vaccine regimens after a 12-week dose interval (15).

Taken together, these data support flexibility in the use of heterologous prime-boost regimens with ChAdOx1 nCoV-19 and BNT162b2. Whether combining other approved vaccines in heterologous prime-boost vaccination strategies also elicit a robust immune response and has a similar reactogenicity profile remains of relevance for immunology and public health policy (14).

The promising data on heterologous prime boost vaccination regimens prompted us to initiate the SWITCH trial. The SWITCH trial is a multi-center, single-blind, randomized controlled trial among Health Care Workers (HCWs) primed with the Ad26.COV2.S vaccine without previous SARS-CoV-2 infection to be executed in four academic hospitals in the Netherlands (ClinicalTrial.gov identifier NCT04927936). The key objective of the SWITCH trial is to compare the effect of a homologous boost with Ad26.COV2.S with a heterologous boost with BNT162.b2 or mRNA1273. To date, Ad26.COV2.S is the only authorized vaccine intended for single vaccination. However, at this moment the safety and efficacy of a two-dose vaccination regimens with a 57-day prime-boost interval is investigated in the ENSEMBLE2 trial (ClinicalTrial.gov identifier NCT04614948). One other heterologous vaccine regimen trial with Ad26.COV2.S is performed by the National Institute of Allergy and Infectious Diseases (NIAID). In this trial, mRNA1273 is used as a boost 12-20 weeks after prime and the effect on SARS-CoV-2 specific antibodies will be evaluated (ClinicalTrial.gov identifier NCT04889209). Two recent studies showed that although there are durable immune responses elicited by a single dose of Ad26.COV2.S (16, 17), boosting may be required to rapidly increase humoral immune responses.

The SWITCH trial (**Figure 1**) started recruitment at the end of June 2021 and will be completed at the end of 2022. HCWs are randomized in one of the four arms: Ad26.COV2.S/no boost; Ad26.COV2.S/Ad26.COV2.S; Ad26.COV2.S/mRNA1273; and Ad26.COV2.S/BNT162b2. The boost will be given 84 days (-7days/+21days) after the prime with Ad26.COV2.S. In total, 432 participants (108 HCWs in each arm) will be randomized. We will analyze the data per protocol, with three pre-defined contrasts, (i) Ad26.COV2.S/no boost *vs.* Ad26.COV2.S/Ad26.COV2.S; (ii) Ad26.COV2.S/Ad26.COV2.S *vs.* Ad26.COV2.S/mRNA1273; and (iii) Ad26.COV2.S/Ad26.COV2.S *vs.* Ad26.COV2.S/BNT162b2. The primary outcome is the detection of SARS-CoV-2 specific antibodies as measured by a quantitative IgG assay 28 days after the boost. Binding antibodies will be measured using the LIAISON SARS-CoV-2 TrimericS IgG assay in combination with the NIBSC/WHO COVID-19 reference serum 20/136 allowing for standardized results (18). Secondary outcomes are reactogenicity and extensive characterization of immunogenicity, including neutralizing antibody titers and SARS-CoV-2-specific T-cell responses against different SARS-CoV-2 variants (also see, (**Supplementary File 1**), page 13). The SWITCH trial was designed in line with the other COVID-19 vaccination randomized controlled trials (10–13) and was approved by the local medical ethics committee (MEC 2021-0132).

**Figure 1:**
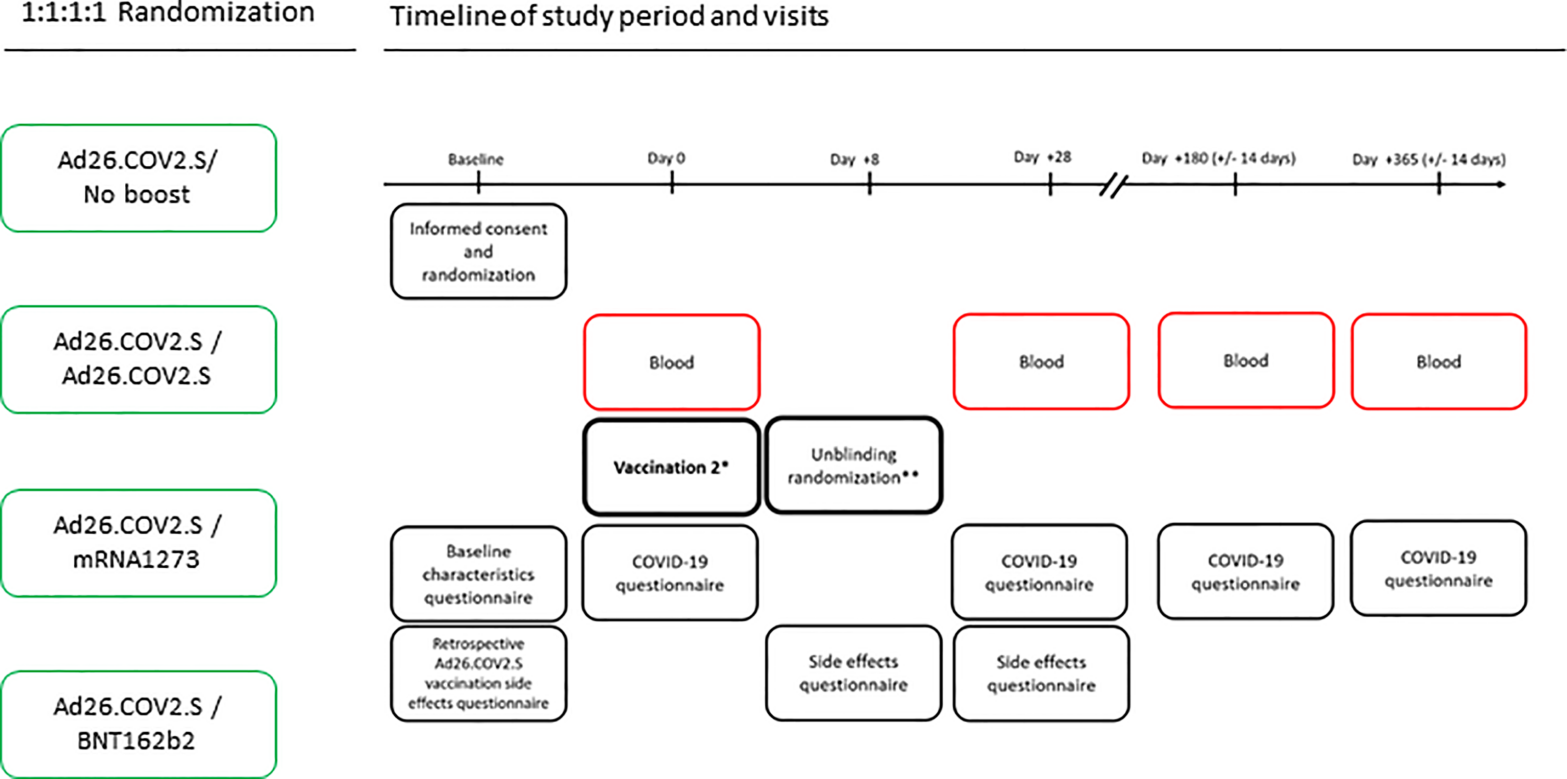
SWITCH trial design. The SWITCH trial includes four arms that receive a booster vaccination 84 days after prime with Ad26.COV2.S. Blood samples will be collected at indicated timepoints by venepunctures. Questionnaires will be performed to collect reactogenicity data and to determine whether participants had breakthrough infections despite vaccinations. *One of the four arms or 25% of the randomized participants will not receive a second vaccination; **Unblinding the randomization of participants will be done after the side effects (i.e., reactogenicity) questionnaire is completed.

This trial will give insight into the immunogenicity and safety of heterologous prime-boost vaccination regimens starting with Ad26.COV2.S, and specifically addresses this knowledge gap in a side-by-side comparison with the homologous counterpart. If booster vaccinations are eventually indicated for individuals who received a single-dose of Ad26.COV2.S, the SWITCH trial will also provide information on which vaccine is best suited as a booster. Reactogenicity data will address the safety profile of heterologous COVID-19 vaccine regimens starting with the Ad26.COV2.S vaccine. In addition to the strength and durability of the immune response, this trial addresses the breadth of the induced immune response by measuring reactivity to circulating SARS-CoV-2 variants. In order to adequately disseminate the results of the SWITCH trial, we will publish the results as soon as possible to enable implementation in vaccination campaign strategy and comparison with other trials.

## Author Contributions

All authors were involved in the design and execution of the study and writing of the manuscript. All authors contributed to the article and approved the submitted version.

## Funding

The trial is funded by ZonMW in the COVID-19 Vaccine program (project grantnumber: 10430072110001).

## Conflict of Interest

The authors declare that the research was conducted in the absence of any commercial or financial relationships that could be construed as a potential conflict of interest.

## Publisher’s Note

All claims expressed in this article are solely those of the authors and do not necessarily represent those of their affiliated organizations, or those of the publisher, the editors and the reviewers. Any product that may be evaluated in this article, or claim that may be made by its manufacturer, is not guaranteed or endorsed by the publisher.

## Supplementary Material

Supplementary File 1 Protocol for the SWITCH trial approved by the medical ethics committee.
