# Supplementary material for: Heterologous Ad26.COV2.S Prime and mRNA-Based Boost COVID-19 Vaccination Regimens: The SWITCH Trial Protocol
Source: Front Immunol. 2021 Sep 24;12:753319. doi: 10.3389/fimmu.2021.753319 (PMC8529966; doi:10.3389/fimmu.2021.753319)
Supplement: Supplementary File 1 — Protocol for the SWITCH trial approved by the medical ethics committee. [file DataSheet_1.pdf]

SWITCH

Switching of COVID-19 Vaccines:
a solution for the problems?

A multicenter, randomised, single-blind, controlled trial among HealthCare Workers (HCW) vaccinated with Janssen: the SWITCH trial.

Switching of COVID-19 Vaccines: a solution for the problems?

**Protocol ID**	MEC-2021-0132<br>NL76782.078.21
**Short title**	SWITCH
**EudraCT number**	2021-000701-24
**Version**	3.1
**Date**	01-06-2021
**Coordinating investigator/project leader**	Drs. Roos Sablerolles<br>Dr. Corine Geurts van Kessel<br>Drs. Maaike Rutten<br>Dr. Rory de Vries
**Principal investigator(s) (in Dutch: hoofdonderzoeker/ uitvoerder)**	**Erasmus MC**<br>Prof. dr. Hugo van der Kuy<br>Dr. Virgil Dalm<br>Dr. Melvin Lafeber<br>Dr. Wim Rietdijk<br>Prof. dr. Marion Koopmans<br><br>**UMCG**<br>Prof. dr. Debbie van Baarle<br>Prof. dr. Anke Huckriede<br>Dr. Douwe Postma<br><br>**AmsterdamUMC**<br>Dr. Bram Goorhuis<br>Prof. dr. Joost Wieringa<br><br>**LUMC**<br>Prof. dr. Leo Visser

**Sponsor (in Dutch: verrichter/opdrachtgever)**	Erasmus MC Rotterdam, the Netherlands
**Subsidising party**	ZonMw (request is being processed)
**Independent expert (s)**	Dr. N.C. Peltenburg<br>n.c.peltenburg@erasmusmc.nl
**Laboratory sites <*if applicable*>**	Viroscience laboratory, Erasmus MC, the Netherlands<br><br>Biochemistry laboratory, Erasmus MC, the Netherlands
**Pharmacy <*if applicable*>**	Hospital Pharmacy, Erasmus MC, The Netherlands

PROTOCOL SIGNATURE SHEET

Name	Signature	Date
**Sponsor or legal representative:**<br>Prof. dr. Hugo van der Kuy<br>Hospital Pharmacist/Clinical Pharmacologist<br>Head of Hospital Pharmacy		25-05-2021
**Coordinating Investigator/Project leader/Principal Investigator:**<br><br>Dr. Virgil Dalm<br>internist-clinical immunologist		25-05-2021

TABLE OF CONTENTS

1. INTRODUCTION AND RATIONALE 11
2. OBJECTIVES 13
Exploratory objective 1. Study the quantity of SARS-CoV-2 specific Tfh cells by IL-21 ELISPOT. 13
3. STUDY DESIGN 14
4. STUDY POPULATION 17
  a. Population (base) 17
  b. Inclusion criteria 17
  c. Exclusion criteria 17
  a. Sample size calculation 18
5. TREATMENT OF SUBJECTS 19
  b. Investigational product/treatment 19
  a. Use of co-intervention (if applicable) 19
  b. Escape medication (if applicable) 19
6. INVESTIGATIONAL PRODUCT 20
7. NON-INVESTIGATIONAL PRODUCT 26
8. METHODS 27
  a. Study parameters/endpoints 27
    Main study parameter/endpoint 27
    Secondary study parameters/endpoints 27
    i. Other study parameters (if applicable) 29
  b. Randomisation, blinding and treatment allocation 29
  c. Study procedures 30
  d. Withdrawal of individual subjects 31
    i. Specific criteria for withdrawal (if applicable) Allergic response to first SARS-CoV-2 vaccination. 31
  e. Replacement of individual subjects after withdrawal 31
  f. Follow-up of subjects withdrawn from treatment 32
  g. Premature termination of the study 32
9. SAFETY REPORTING 33
  h. Temporary halt for reasons of subject safety 33
  i. AEs, SAEs and SUSARs 33
    i. Adverse events (Aes) 33
    ii. Adverse Reaction (AR) 33
    iii. Serious adverse events (SAEs) 33

iv. Suspected unexpected serious adverse reactions (SUSARs) 34
j. Annual safety report 36
k. Follow-up of adverse events 36
l. [Data Safety Monitoring Board (DSMB) / Safety Committee] 36
10. STATISTICAL ANALYSIS 38
a. Primary study parameter(s) 39
b. Secondary study parameter(s) 39
c. Other study parameters 39
d. Interim analysis (if applicable) 39
11. ETHICAL CONSIDERATIONS 40
a. Regulation statement 40
b. Recruitment and consent 40
c. Objection by minors or incapacitated subjects (if applicable) 41
d. Benefits and risks assessment, group relatedness 41
e. Compensation for injury 42
f. Incentives (if applicable) 43
12. ADMINISTRATIVE ASPECTS, MONITORING AND PUBLICATION 44
a. Handling and storage of data and documents 44
i. Confidentiality of the data 44
ii. Confidentiality of subject records 44
iii. Data Management 45
iv. Biological samples 45
b. Monitoring and Quality Assurance 45
c. Amendments 46
d. Annual progress report 46
e. Temporary halt and (prematurely) end of study report 46
f. Public disclosure and publication policy 46
13. STRUCTURED RISK - BENEFIT ANALYSIS 47
14. REFERENCES 48

LIST OF ABBREVIATIONS AND RELEVANT DEFINITIONS

ABR	General Assessment and Registration form (ABR form), the application form that is required for submission to the accredited Ethics Committee; in Dutch: Algemeen Beoordelings- en Registratieformulier (ABR-formulier)
AE	Adverse Event
AR	Adverse Reaction
CA	Competent Authority
CCMO	Central Committee on Research Involving Human Subjects; in Dutch: Centrale Commissie Mensgebonden Onderzoek
CV	Curriculum Vitae
DSMB	Data Safety Monitoring Board
EU	European Union
EudraCT	European drug regulatory affairs Clinical Trials
GCP	Good Clinical Practice
GDPR	General Data Protection Regulation; in Dutch: Algemene Verordening Gegevensbescherming (AVG)
HCW	HealthCare Workers
IB	Investigator’s Brochure
IC	Informed Consent
IMP	Investigational Medicinal Product
IMPD	Investigational Medicinal Product Dossier
METC	Medical research ethics committee (MREC); in Dutch: medisch-ethische toetsingscommissie (METC)
(S)AE	(Serious) Adverse Event
SPC	Summary of Product Characteristics; in Dutch: officiële productinformatie IB1-tekst
Sponsor	The sponsor is the party that commissions the organisation or performance of the research, for example a pharmaceutical company, academic hospital, scientific organisation or investigator
SUSAR	Suspected Unexpected Serious Adverse Reaction
UAVG	Dutch Act on Implementation of the General Data Protection Regulation; in Dutch: Uitvoeringswet AVG
WMO	Medical Research Involving Human Subjects Act; in Dutch: Wet Medisch-wetenschappelijk Onderzoek met Mensen

SUMMARY

Rationale: A novel coronavirus (SARS CoV-2) was first detected in Wuhan, China in December 2019 (1). In January 2021 the first vaccines protecting against this virus are available in the Netherlands. Most current available vaccines in the Netherlands (e.g., AstraZeneca(2), Moderna(3), and Pfizer(4)) require a second boost (2nd vaccination) after the prime (1st vaccination), to obtain an optimal immune response. The booster (2nd vaccination) has up to present always been given with the same vaccine as the primary vaccine. Unfortunately, the availability of the vaccines is limited and a speedy vaccination is also hampered by logistic reasons. The ability to combine different vaccines could make vaccination programs in the future more flexible. It would facilitate a fast-track process and reduce the impact of any supply-chain disruptions (5). In addition, several studies in mice have already shown that combining different vaccines (two-dose heterologous vaccination regimen) can elicit a broader immune response (in field of neutralizing antibodies and T-cell responses)(6, 7). These results endorse the need for clinical trials to investigate the immunogenicity of heterologous regimens. Trails to combine different vaccines have meanwhile been set up in Great Britain and Spain where they combine Pfizer and AstraZeneca (Com-COV 1(8) and CombiVacS(9)). Recently, we were confronted that the CoM-COV1 was expanded with Moderna and Novavax (CoM-COV2(10)). Therefore we focus our protocol to adeno priming with Janssen, which is not studied in the other ongoing studies. As the vaccination rate in The Netherlands is increasing rapidly, we decided to include Healthcare Workers (HCW) vaccinated once with Janssen.

Objective: The key objective of the study is to measure the immune response against SARS-CoV-2 after different vaccinations in Health Care Workers (HCW) from 18 to 65 years old.
Determination of antibodies by a quantitative IgG assay (LIAISON SARS-CoV-2 TrimericS IgG essay) 28 days after second vaccination (booster) comparing, per protocol, the following three groups:

a. Janssen/- vs. Janssen/Janssen
b. Janssen/Janssen vs. Janssen/Pfizer
c. Janssen/Janssen vs. Janssen/Moderna

Study design: A multicenter, randomised, single-blind, controlled trial to determine reactogenicity and immunogenicity of different prime-boost COVID-19 vaccine schedules.

Setting: multicenter study conducted at 4 academic university hospitals (Amsterdam UMC, Erasmus MC, Leiden UMC, and UMC Groningen).

Hypothesis: Immunogenicity in participants who have already received one dose of Janssen will be higher when it is followed by a heterologous booster containing Pfizer or Moderna as opposed to a homologous booster containing Janssen.

The main question that will be addressed: Measuring the humoral immune response against SARS-CoV-2 after inoculation with a single-dose Janssen compared to a homologous vaccination regimen with Janssen/Janssen and the comparison of a homologous vaccination regimen (Janssen/Janssen) with a heterologous vaccination regimen (Janssen/Pfizer + Janssen/Moderna).

Participants will be randomized for Standard of Care (1 vaccination with Janssen), a homologue vaccination strategy (two vaccines from the same manufacturer, i.e., Janssen Pharmaceutica) or a heterologous vaccination strategy (two different vaccines, e.g., Janssen followed by Pfizer or Moderna).

The study starts approximately 84 days (+/- 10) after the first vaccination Janssen. The day of the 2nd vaccination is seen as day 0.

Blood will be drawn at 4 different time points, i.e. day 0 - baseline (before 2nd vaccination), day 28 (after 2nd vaccination - primary endpoint), day 180 +/- 14 days (after 2nd vaccination), and day 365 +/- 14 days (after 2nd vaccination). Questionnaires will be used to monitor for adverse reactions after 2nd vaccination and to evaluate COVID-19 infection during the study and outcome despite vaccination.

Study population: Healthcare Workers (HCW) from 18 to 65 years old vaccinated once with Janssen. Individuals of all ethnicities will be recruited. Given the speed of the Dutch vaccination campaign, it is not feasible to collect baseline immunological data before first vaccination. For this reason, the baseline in this study is at the day of the booster (2nd vaccination). If we cannot recruit enough HCW within the recruiting hospitals, we expand our population to HCW in surrounding peripheral hospitals and primary care (e.g., local pharmacies, dental practices, and physiotherapists).

Intervention (if applicable): All adult people in the Dutch population are vaccinated on a voluntary basis. The only difference is that they might be vaccinated with a different vaccine for the boost (second vaccination).

Main study parameters/endpoints:

<u>Primary endpoint</u>: to determine whether the immune response 28 days after boost is higher to that observed following only one vaccination (Janssen/- vs. Janssen/Janssen) and the comparison of a heterologous boost of a COVID-19 vaccine (84 days post prime (1st vaccination) – Janssen/Pfizer and Janssen/Moderna) with a homologous boost (84 days post prime (1st vaccination) – Janssen/Janssen), in participants vaccinated once with Janssen.

Secondary endpoints: to assess safety & reactogenicity of different prime-boost schedules of COVID-19 vaccines and characterization of immunogenicity of different prime-boost schedules of COVID-19 vaccines.

Nature and extent of the burden and risks associated with participation, benefit and group relatedness: The consequence of participating in this study has two sides; on the one hand, a higher burden for the participant, given 4 study visits in one year including blood samples and questionnaires. With the fact that slightly more side effects may be seen in the first 48 hours after the second vaccination (11). In contrast, in a prospective study from Germany with the same vaccination strategy, they identified a comparable reactogenicity between heterologous (Astra Zeneca/Pfizer) and homologous (Pfizer/Pfizer) booster vaccination after a 12-week dose interval (12). On the other hand, participants receive an overview of their own immune response after vaccination at several time points and their participation contributes to answering a very relevant research question. We expect the burden of the study visits to be minimal.

1. INTRODUCTION AND RATIONALE

The novel coronavirus (SARS CoV-2) was first detected in Wuhan, China in December 2019(1). In January 2021, multiple vaccines against COVID-19 were approved and became available for emergency use in the Netherlands.

Most current available vaccines in the Netherlands (e.g., AstraZeneca (2), Moderna (3), and Pfizer (4)) require a second boost after primary vaccination, to obtain an optimal immune response. The booster vaccination has up to present always been given with the same vaccine as the primary vaccine. Unfortunately, the availability of vaccines is limited and a speedy vaccination is also hampered by logistic reasons. There would be advantages to having flexible immunization programs where the second dose is not necessarily the same as the first dose, i.e. a permissive approach to using heterologous boost schedules. It would facilitate a fast-rack process and reduce the impact of any supply-chain disruptions (5).
In addition, several studies in mice have already shown that combining vaccinations (two-dose heterologous vaccination regimen) can elicit a broader immune response (in field of neutralizing antibodies and T-cell responses) (6, 7).

These results endorse the need for clinical trials to investigate the immunogenicity of heterologous vaccination regimens.
Trails to combine different vaccinations have meanwhile been set up in Great Britain (Com-COV (8)) and Spain (CombiVacS (9)). The University of Oxford combine AstraZeneca and Pfizer and vice versa in adults over 50 years of age (Com-COV study (8)). Recently, they expanded their trial with Moderna and NovaVax in the Com-COV2 (10) study were patients over 50 already had their first vaccination with either AstraZeneca or Pfizer, followed by a boost with Moderna, Pfizer, AstraZeneca or Novavax. The study starts with blood samples at vaccination 2. The CombiVacS (9) examine the safety and immunogenicity in adult subjects (18 years and older) in good health or stable clinical situation who have already received one dose of AstraZeneca followed by one dose of Pfizer 8-12 weeks later. In line with these studies, blood samples start with vaccination 2 in our trial too. This to enhance comparability across studies.

Based on the trials that already have started, we have decided to focus on Janssen in this study. The Janssen vaccine is currently registered as a single shot vaccine, reported to be 66% effective in preventing moderate to severe COVID-19 (13). Nevertheless, a second vaccine may result in an increased and more durable immune response. Hence, Janssen has already initiated a phase 3 clinical trial to evaluate the efficacy and safety of a 2-dose

regimen (with an interval for 57 days), the ensemble 2 study (14). It is expected that a boost with Janssen would be the new Standard of Care (SoC) within the next months.

Therefore we have decided to study both a single-regimen, and a two-dose regimen with homologous and heterologous boost schedules. Janssen is used for the first vaccination (prime), followed by nothing or Janssen, Moderna or Pfizer as a second vaccination (boost). If we do not include the newly expected SoC, our study can no longer be used as a comparison with the new SoC and thus cannot be applied for implementation. In addition, it is difficult to calculate a sample size without a control arm with a homologous boost. A two dose regimen compared to a single dose always produces a higher and better immune response. Therefore, to understand the immunological characteristics of the boost response (breadth of the neutralizing repertoire, and T-cell responses next to height GMT VNT), an arm with an homologous boost is needed. Second, a two-dose regimen will likely be required to provide adequate protection against virus variants. The crucial importance of a second vaccination was recently demonstrated for the Indian variant B.1.617.2 in the UK. A single immunization with either Pfizer or AstraZeneca vaccine provided only 33% protection against symptomatic disease. A second immunization increased protection to 88% and 66% for the Pfizer and AstraZeneca vaccine respectively (15). Finally, we opt for an extended interval of 12 weeks between the two doses, an extended interval was shown to have the greatest impact on the efficacy of the vaccine in a study performed with the AstraZeneca vaccine (16, 17) and is used in both Com-COV and CombiVacS.

With this trial, we aim to build upon the recently initiated studies (Com-COV and CombiVacS) and suggest to perform a randomized controlled trial comparing a homologous and heterologous vaccination strategy. Given the speed of the Dutch vaccination campaign, it is not feasible to collect baseline immunological data. Our study, in line with the Com-COV2 and the CombiVacS, will start on day of boost (second vaccination; i.e., 84 days after first vaccination).

2. OBJECTIVES

Primary objective

The key objective of the study is to measure the humoral immune response against SARS-CoV-2 28 days after different vaccination strategies in Health Care Workers (HCW).

Determination of antibodies by a quantitative IgG assay (LIAISON SARS-CoV-2 TrimericS IgG essay) 28 days after second vaccination (booster) comparing the three following groups:

d. Janssen/- vs. Janssen/Janssen
e. Janssen/Janssen vs. Janssen/Pfizer
f. Janssen/Janssen vs. Janssen/Moderna

Secondary objectives

1. To assess safety of homologous/heterologous prime-boost schedules of COVID-19 vaccines with Janssen/Moderna/Pfizer.
Outcome measures; (serious) adverse events during the study.

2.Reactogenicity and safety of homologous/heterologous prime-boost schedules of COVID-19 vaccines with Janssen/Moderna/Pfizer.
Outcome measures; solicited local & systemic reactions 7 days after boost (2nd vaccination). Changes from baseline in laboratory safety measures (Day 0, Day 28).

3.Further characterization of immunogenicity of homologous/heterologous prime-boost schedules of COVID-19 vaccines with Janssen/Moderna/Pfizer.
Outcome measures; antibodies/neutralizing antibodies, quantity of SARS-CoV-2 specific T-cells by IFNy ELISPOT, in-depth functional and phenotypical characterization of SARS-CoV-2 specific T-cells by flow cytometric analyses, and longevity of SARS-CoV-2 specific T-cells (Day 0, Day 28, Day 180 +/- 14 days, Day 365 +/-14 days).

4. Evaluation of immunogenicity, safety, and reactogenicity of COVID-19 vaccines in participants sero-positive (determined with nucleocapsid (N)-specific antibody ELISA's) for SARS-CoV-2 IgG at baseline.
Outcome measures; Immunogenicity, reactogenicity, and safety endpoints as outlined above.

Exploratory objective

1. Study the quantity of SARS-CoV-2 specific Tfh cells by IL-21 ELISPOT.

*All immunogenicity outcomes will be measured in 50% of the participants.

3. STUDY DESIGN

A multicenter, randomised, single-blind, controlled trial to determine reactogenicity and immunogenicity of different prime-boost COVID-19 vaccine schedules.

Setting

Multicenter study conducted through 4 academic trial sites (Amsterdam UMC, Erasmus MC, Leiden UMC, and UMC Groningen).

Trial duration

Total duration of each participant will be 12 months from the administration of the first vaccine dose.

Study groups

This study will consists of 1 cohort (n=108 per arm) of which half of the participants will undergo a detailed immunological assessment (n=54 per arm).

Group	Vaccination 1 Day -84 not in study	Vaccination 2 day 0	Number of participants	PBMC
J/M	Janssen	Moderna	108	54
J/P	Janssen	Pfizer	108	54
J/J	Janssen	Janssen	108	54
J	Janssen	-	108	54

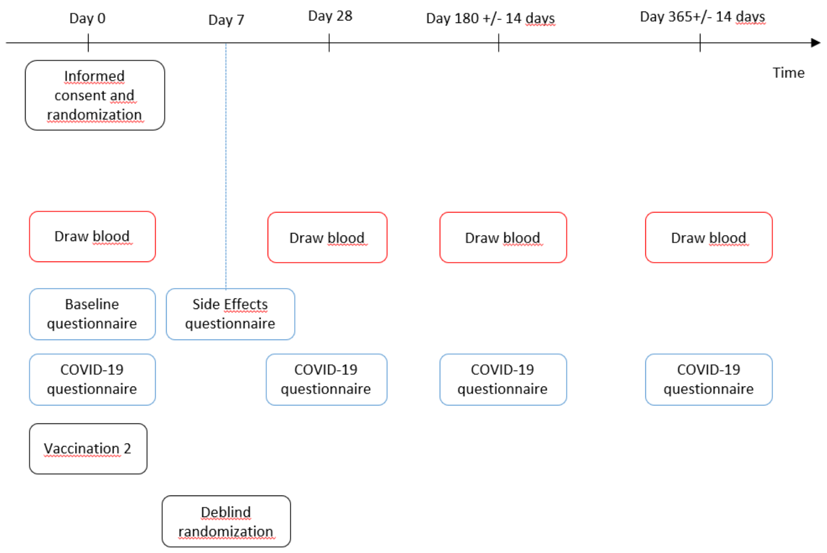

Randomisation/Blinding

Participants will be randomised per cohort in 1:1:1:1 fashion using block randomisation.
The study will be single-blind. Staff involved in study will be aware of which vaccine the participant is receiving (arm allocation); the participants will remain blinded to their vaccine allocation.
Vaccines will be prepared in the hospital pharmacy unit where each vaccine is coded as a letter. Laboratory staff will also be blinded to the vaccine schedule received.
Seven days (after filling in the questionnaires regarding side-effects) after the boost (2nd vaccination) the vaccination strategy will be unblinded. Directly after the boost (2nd vaccination) a letter will be given in which the study is explained and a statement is given that a second vaccination is given. The registration card will be supplied 7 days after the boost (2nd vaccination).
Those participants receiving only one shot with Janssen will only be told on the day of planned boost (2nd vaccination). They will not be vaccinated with placebo. Even though these participants will not receive a boost (2nd vaccination), they will be asked to complete the questionnaires and blood will be drawn at all given time points.
If the clinical condition of a participant necessitates unblinding of the participant, this will be done if unblinding is thought to be relevant and likely to change clinical management.

4. STUDY POPULATION

a. Population (base)

Healthcare Workers (HCW) from 18 to 65 years old vaccinated once with Janssen. Individuals of all ethnicities will be recruited. Given the speed of the Dutch vaccination campaign, it is not feasible to collect baseline immunological data before first vaccination. For this reason, the baseline in this study is at the day of the booster (2nd vaccination). If we cannot recruit enough HCW within the recruiting hospitals, we expand our population to HCW in surrounding peripheral hospitals and primary care (e.g., local pharmacies, dental practices, and physiotherapists).

b. Inclusion criteria

1. Participant is willing and able to give written informed consent for participation in the trial.
2. Adult (male/female) between 18 and 65 years old
3. Sufficient level of the Dutch language to undertake all study requirements

c. Exclusion criteria

1. Adults younger than 18 or older than 65 years
2. Adults already vaccinated with other vaccine then Janssen
3. Previously had a COVID-19 infection
4. History of allergic reaction likely to be exacerbated by any component of study vaccines (e.g. hypersensitivity to the active substance or any of the SmPC-listed ingredients of the Janssen/Pfizer/Moderna vaccine).
5. Adults that are pregnant or have a wish to become pregnant within 6 months
6. Currently being treated for cancer
7. Severe kidney failure or dialyses dependent
8. Status after organ-, stem cell- or bone marrow transplantation
9. Use of immunosuppressant's
10. Epilepsy
11. HIV
12. Bleeding disorder (e.g. factor deficiency, coagulopathy or platelet disorder), or prior history of significant bleeding of bruising following IM injections of vene puncture
13. Continuous use of anticoagulants, such as coumarins (e.g. acenocoumarol) or novel oral anticoagulants (i.e. apixaban, dabigatran etc).

14. Participants who are currently participating in another research trial.
15. All regular contra-indications of the vaccines will be applied

a. Sample size calculation

The primary analysis of this study will consist of three comparisons:

- Jansen/- vs Janssen/Janssen
- Janssen/Janssen vs Janssen/Pfizer
- Janssen/Janssen vs Janssen/Moderna

The primary endpoint is the antibody response on day 28 after the boost (2nd vaccination).

The sample calculation is based on the following assumptions:

- The sample size calculation takes the available data from the Com-CoV study. We are aware that these characteristics are derived from the AstraZeneca vaccines, but no data is available for Janssen. However, similar distribution of the immune response is expected as we aim to include healthy HCW too. At the same time, we took the primary endpoint and study design from the CombiVacs study, as we closely mimick their protocol. In addition, we aim to test whether boosting with a different vaccine as compared to the first vaccination (e.g. moderna or Pfizer) is superior in eliciting an immune response to boosting with the same vaccine (i.e. Janssen).
- The Geometric Mean Concentration (GMC) differences between two groups in log base 10 of titer determination based on the Com-CoV protocol are around 500 EU/ml, log base 10, 2.69 and 2.50; Where the mean difference between the two groups in the three comparisons is estimated to be 8%.
- The standard deviation of GMC on log scale (log base 10) is 0.4 (similar assumptions underlying immune response distributions to the Com-CoV trial).

Based on the above assumptions, the study will need to recruit 85 patients who are seronegative for SARS-CoV-2 IgG at baseline in each arm to achieve 80% power at one-sided 2.5% significance level. We assume +/- 25% of study participants will be excluded from the per protocol analysis due to seropositive for SARS-CoV-2 IgG at baseline or loss to follow-up. Therefore, the sample size in each arm will be 108 participants. The study contains four treatment arms resulting in a total sample size of 432 participants.

5. TREATMENT OF SUBJECTS

Vaccines against SARS-CoV-2 are standard of care in the Netherlands. This study will investigate the immune response and AEs after both standard care and if a different vaccination strategy is followed after the first boost. If subjects did not participate in this study, they would also receive one of the registered vaccines via the RIVM distributions pathways.

b. Investigational product/treatment

All participants were vaccinated once with Janssen. Then the participants will be divided into 4 groups; Standard of Care (SoC - only once Janssen) or a booster with either, Janssen/Moderna or Pfizer (2nd vaccine). All vaccines are given into the muscle of the upper arm 84 days apart. The vaccines will come directly from RIVM and are not part of the stock used for the national vaccination program. These vaccinations are reserved by RIVM for research purposes. All used vaccines are registered COVID-19 Vaccines.

a. Use of co-intervention (if applicable)

Not applicable

b. Escape medication (if applicable)

Not applicable

6. INVESTIGATIONAL PRODUCT

The vaccines that are used are officially registered by the EMA.

Vaccine	Registration date + Link SmPC
Pfizer	23/12/2021<br>Clean COVID-19 Vaccine SmPC-PL 21-DEC-2020 735AM EST-135PM CET (europa.eu)
Moderna	06/01/2021<br>COVID-19 Vaccine Moderna, Common name-COVID-19 mRNA Vaccine (nucleoside modified) (europa.eu)
Janssen Pharmaceutica	11/03/2021<br>Covid-19 vaccine Janssen 5737 - PI_clean_EN (europa.eu)

a. Name and description of non-investigational product(s)

Vaccine	Description
Pfizer	Comirnaty contains a molecule called messenger RNA (mRNA) with instructions for producing a protein from SARS-CoV-2, the virus that causes COVID-19. Comirnaty does not contain the virus itself and cannot cause COVID-19.
Moderna	Moderna contains a molecule called messenger RNA (mRNA) with instructions for producing a protein from SARS-CoV-2, the virus that causes COVID-19. Comirnaty does not contain the virus itself and cannot cause COVID-19.
Janssen Pharmaceutica	COVID-19 Vaccine Janssen works by preparing the body to defend itself against COVID-19. It is made up of another virus (an adenovirus) that has been modified to contain the gene for making the SARS-CoV-2 spike protein. This is a protein on the SARS-CoV-2 virus which it needs to enter the body's cells.

	The adenovirus passes the SARS-CoV-2 gene into the vaccinated person's cells. The cells can then use the gene to produce the spike protein. The person's immune system will recognise the spike protein as foreign and produce antibodies and activate T cells (white blood cells) to target it.<br>Later, if the person comes into contact with SARS-CoV-2 virus, the vaccinated person's immune system will recognise the spike protein on the virus and be ready to defend the body against it.<br>The adenovirus in the vaccine cannot reproduce and does not cause disease.

b. Summary of findings from non-clinical studies

Not applicable. All vaccines are registered.

c. Summary of findings from clinical studies

Vaccine	Clinical findings
Pfizer	43.548 patients<br>A two-dose regimen of BNT162b2 conferred 95% protection against Covid-19 in persons 16 years of age or older. Safety over a median of 2 months was similar to that of other viral vaccines.<br>Safety and Efficacy of the BNT162b2 mRNA Covid-19 Vaccine - PubMed (nih.gov)
Moderna	30.000 patients<br>Conclusions: The mRNA-1273 vaccine showed 94.1% efficacy at preventing Covid-19 illness, including severe disease. Aside from transient local and systemic reactions, no safety concerns were identified.<br>Efficacy and Safety of the mRNA-1273 SARS-CoV-2 Vaccine - PubMed (nih.gov)

Janssen Pharmaceutica	40.000 patients<br>The effectiveness data to support the EUA include an analysis of 39,321 participants in the ongoing randomized, placebo-controlled study being conducted in South Africa, certain countries in South America, Mexico, and the U.S. who did not have evidence of SARS-CoV-2 infection prior to receiving the vaccine. Among these participants, 19,630 received the vaccine and 19,691 received placebo. Overall, among these clinical trial participants, the vaccine was approximately 67% effective in preventing moderate to severe/critical COVID-19 disease occurring at least 14 days after vaccination and 66% effective in preventing moderate to severe/critical disease at least 28 days after vaccination.<br>Janssen COVID-19 Vaccine Frequently Asked Questions | FDA

d. Summary of known and potential risks and benefits

<u>Potential risks from vaccine administration (Janssen / Moderna / Pfizer).</u>

Hypersensitivity to the active substance or to any of the excipients.

The most common side effects with all these vaccines are usually mild or moderate and got better within a few days after vaccination. Local and systemic adverse reactions were reported more frequently after dose 2 than after dose 1.

Very common (>10%): pain and/or swelling at the injection site, lymphadenopathy (axillary on the same side as the injection site), headache, nausea, vomiting, muscle and joint pain, fatigue, chills, and fever.

Common (1-10%): (itchy)rash or redness at the injection side. Skin rash.

Uncommon (0.1-1%): itching at the injection side, insomnia, malaise, pain in extremities, tremor, sneezing, sore throat, hyperhidrosis, muscle weakness, backache, and asthenia.

Rare (0.01-0.1%): hypersensitivity (allergic reaction of skin and underlying tissue), urticaria.

Acute peripheral facial paralysis or palsy (in reports seen from day 22 to day 32 after the 2nd dose with Moderna and from day 3 to 48 after vaccination with Pfizer.

Facial swelling reported in individuals who have had facial dermal fillers in the past (Moderna).

Very rare (<0.01%): thrombosis in combination with thrombocytopenia; includes venous thrombosis such as cerebral venous sinus thrombosis, splanchnic venous thrombosis and arterial thrombosis.
Futhermore, anaphalyaxis, hypersensitivity have been reported.
As for all vaccines, the vaccines will be given under close supervision with appropriate medical treatment available.

Increased reactogenicity from heterologous prime/boost immunisation schedules
Recently, an interim analysis of participants from the com-CoV study has shown that immunization with heterologous schedules of Astra Zeneca and Pfizer result in more frequent solicited systemic reactions such as fatigue, chills, feverishness and malaise than the homologous schedules for these vaccines. They reported the experienced systemic reactions in the first 48 hours after booster vaccination and were not associated with hospital admissions (11). In contrast, in a prospective study from Germany with the same vaccination strategy, they identified a comparable reactogenicity between heterologous (Astra Zeneca/Pfizer) and homologous (Pfizer/Pfizer) booster vaccination after a 12-week dose interval (12). The heterologous combination from our study will have to reveal what the exact effect is on reactogenicity.

Potential benefits
These vaccines offer a high level of protection against COVID-19 which is a critical need in the current pandemic (see table for efficacy rates). Most side effects are mild to moderate in severity and are gone within a few days. The Agency therefore decided that these vaccines benefits are greater than its risks and that they can be authorized for use in the EU. All vaccines have been granted a conditional marketing authorization. This means that there is more evidence to come about the vaccine, which the company is required to provide. The Agency will review any new information that becomes available and this overview will be updated as necessary.

**Vaccine**	**Efficacy**
Pfizer	up to 95%
Moderna	up to 95%
Janssen Pharmaceutica	The efficacy is approximately 67% effective in preventing moderate to severe/critical COVID-19 disease occurring at least 14 days

	after vaccination and 66% effective in preventing moderate to severe/critical disease at least 28 days after vaccination. Additionally, the vaccine was approximately 77% effective in preventing severe/critical COVID-19 occurring at least 14 days after vaccination and 85% effective in preventing severe/critical COVID-19 occurring at least 28 days after vaccination.

e. Description and justification of route of administration and dosage

All vaccines will be administered according to their respectively SmPC registration.
Also the dosage will be administered according to the SmPC.
All vaccines are given as two injections, usually into the muscle of the upper arm, at least 84 days apart.

f. Dosages, dosage modifications and method of administration

**Vaccine**	**Dosage**
Pfizer	0.3 ml IM after dilution with NaCl 0.9%
Moderna	0.5 ml IM
Janssen Pharmaceutica	0.5 ml IM

g. Preparation and labelling of Non Investigational Medicinal Product

**Vaccine**	**Preparation**
Pfizer	The thawed vaccine must be diluted in its original vial with 1.8 mL sodium chloride 9 mg/mL (0.9%) solution for injection, using a 21 gauge or narrower needle and aseptic techniques.<br>Gently invert the diluted dispersion 10 times. Do not shake.

	Withdraw 0.3 mL of Comirnaty.
Moderna	Withdraw 0.5 mL of Moderna.
Janssen Pharmaceutica	Withdraw 0.5 mL of Janssen Pharmaceutica

h. Drug accountability

The vaccines will be supplied by the RIVM after approval by ZonMw and VWS. It is important to state that the vaccines for this study will NOT be retrieved from a stock reserved for patients or other persons.

As is usual in routine care at this moment, the batch numbers will be recorded for each patients ensuring its traceability. Vaccines will be shipped to the pharmacy. A drug accountability form will be used to record IMP dispensing.

7. NON-INVESTIGATIONAL PRODUCT

This paragraph is not applicable. Only vaccines will be used described in paragraph 6.

SWITCH

8. METHODS

a. Study parameters/endpoints

Main study parameter/endpoint

The key objective of the study is to measure the humoral immune response against SARS-CoV-2 28 days after different vaccination strategies in Health Care Workers (HCW) vaccinated once with Janssen.

Determination of antibodies by a quantitative IgG assay (LIAISON SARS-CoV-2 TrimericS IgG essay) 28 days after second vaccination (booster) comparing, per protocol, the following three groups:

a. Janssen/- vs. Janssen/Janssen
b. Janssen/Janssen vs. Janssen/Pfizer
c. Janssen/Janssen vs. Janssen/Moderna

Antibodies will be determined by a quantitative IgG assay (LIAISON® SARS-CoV-2 TrimericS IgG assay). In the Dutch harmonization initiative this assay has been compared to all other platforms which are currently used in the Dutch ZonMw vaccination studies (i.e. PRNT50, pseudo virus assays, Luminex, Wantai ELISA, Sanquin in-house ELISA) and has a very good performance.

Secondary study parameters/endpoints

1. To assess safety of homologous/heterologous prime-boost schedules of COVID-19 vaccines with Janssen/Moderna/Pfizer.
Outcome measures; (serious) adverse events during the study.

2.Reactogenicity and safety of homologous/heterologous prime-boost schedules of COVID-19 vaccines with Janssen/Moderna/Pfizer.
Outcome measures; solicited local & systemic reactions 7 days after immunization (prime as booster). Changes from baseline in laboratory safety measures (Day 0, Day 28)

<u>Laboratory safety measures</u>
Haematology – full blood count
Biochemistry – sodium, potassium, urea, creatinine, albumin, liver function tests (ALT, ALP, bilirubin) and if relevant C-reactive protein (CRP)

3.Further characterization of immunogenicity of homologous/heterologous prime-boost schedules of COVID-19 vaccines with Janssen/Moderna/Pfizer (Day 0, Day 28, Day 180 +/-14 days, Day 365 +/-14 days).

Outcome measures;
- Antibodies (LiaisonTrimericS IgG assay).
- Nucleocapsid (N)-specific antibody ELISA's to determine whether study participants previously experienced COVID-19 (Day 0).
- Neutralizing antibodies (via a validated virus neutralization assay (PRNT50)(18-22).
- Quantity of SARS-CoV-2 specific T-cells by IFNy ELISPOT. To this end, PBMC will be thawed and stimulated with commercially available SARS-CoV-2 peptide pools (JPT peptide technologies, as harmonized to other ZonMw studies, including proper controls). Production of IFNɣ will be detected on ELISPOT plates and expressed as the number of SARS-CoV-2-specific T-cells / million PBMC (mean of 3 measures).
- In-depth functional and phenotypical characterization of SARS-CoV-2 specific T-cells by flow cytometric analyses. These analyses will be performed on a subset of samples, based on the IFNɣ ELISPOT results. To this end, PBMC will be thawed and stimulated with SARS-CoV-2 peptide pools (JPT peptide technologies, as harmonized to other ZonMw studies, including proper controls). After a 24-hour incubation period, PBMC will be acquired by flow cytometry and SARS-CoV-2-specific T-cells will be detected by the specific upregulation of activation markers OX40, CD69 and CD137 (22, 23). Additionally, SARS-CoV-2-specific T-cells will be further phenotyped as CD4+ or CD8+, in combination with phenotypic cell surface T-cell markers to distinguish memory subsets like CD45RA and CCR7.
- Longevity of SARS-CoV-2 specific T-cells
Assays described above will be performed on a long-term sample from study participants acquired 180 and 365 days post boost-vaccination to study the longevity and durability of vaccine-induced T-cells.

4. Evaluation of immunogenicity, safety & reactogenicity of COVID-19 vaccines in participants sero-positive for SARS-CoV-2 IgG at baseline.
Outcome measures; safety, reactogenicity & immunogenicity endpoints as outlined above.

Exploratory objective

1. Since production of antibodies is dependent on follicular T helper cells (Tfh), we will study the quantity of SARS-CoV-2-specific Tfh cells by IL-21 ELISPOT. To this end, PBMC will be thawed and stimulated with commercially available SARS-CoV-2 peptide pools (JPT peptide

technologies, as harmonized to other ZonMw studies, including proper controls). Production of IL-21 will be detected on ELISPOT plates and expressed as the number of SARS-CoV-2-specific T-cells / million PBMC (mean of 3 measures).

*All immunogenicity outcomes will be measured in 50% of the participants.

i. Other study parameters (if applicable)

For the analysis of the data the study parameters which are collected are gender, age, co-medication, comorbidity, and actual COVID-19 infections.

b. Randomisation, blinding and treatment allocation

The cohort will be filled by Amsterdam UMC (n=80), LUMC (n=80), UMCG (n=80) and Erasmus MC (n=192) with 432 participants vaccinated once with Janssen. Participants that will be vaccinated will be randomised in one of the following groups.

- No boost
- Boost with Janssen
- Boost with Moderna
- Boost with Pfizer

Randomization will be done after obtaining written informed consent. Seven days after the boost (2nd vaccination) the vaccinations will be unblinded. Those participants receiving only one shot with Janssen will only be told on the day of planned boost (2nd vaccination). They will not be vaccinated with placebo. On this appointment, only blood is taken. In the rest of the study, the participants follow exactly the same research procedures as the other groups.

SAMPLING STRATEGY

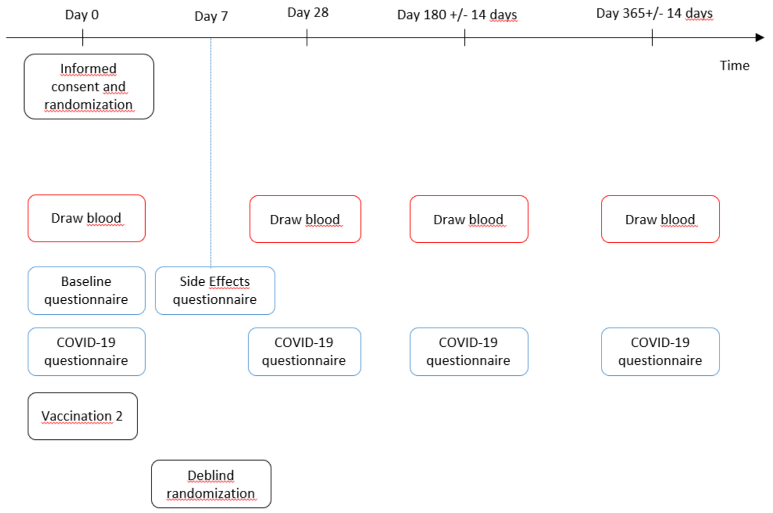

c. Study procedures

All participants at day 0 (day of boost/2nd vaccination):

- One EDTA tube (4 ml)
- One heparin tube (5.5ml)
- One citrate tube (3ml)
- One serum tube (8.5ml)
- Four lithium heparin tubes (4x10ml)*
- Boost with vaccine 2 (if applicable**)
- Request to fill a baseline characteristics questionnaire and COVID-19 questionnaire (online questionnaires via e-mail).

*only in the detailed immunological assessment group

**except for the Janssen/- group

All participants after 7 days (day 7)

- Request to fill a side effects questionnaire (solicited local & systemic reactions)

All participants after 28 days (day 28)

- One EDTA tube (4ml)
- One heparin tube (5.5ml)
- One citrate tube (3ml)
- One serum tube (8.5ml)
- Four lithium heparin tubes (4x10ml)*
- Request to fill a COVID-19 questionnaire

*only in the detailed immunological assessment group
**except for the Janssen/- group

All participants 180 days after 2nd vaccination (day 180)

- One serum tube (8.5ml)
- Four lithium heparin tubes (4x10ml)*
- Request to fill a COVID-19 questionnaire

*only in the detailed immunological assessment group

All participants 365 days after 2nd vaccination (day 365)

- One serum tube (8.5 ml)
- 4 lithium heparin tubes (4x10ml)*
- Request to fill a COVID-19 questionnaire

*only in the detailed immunological assessment group

Questionnaires

At the start of the study, a questionnaire about the basic characteristics of the HCW follows. Seven days after boost (2nd vaccination) one questionnaire will be used to monitor side effects of the vaccine. At each visit one additional questionnaire will be used to monitor occurrence and outcome of COVID-19 infection throughout the study.

d. Withdrawal of individual subjects

Subjects can leave the study at any time for any reason if they wish to do so without any consequences. The investigator can decide to withdraw a subject from the study for urgent medical reasons.

i. Specific criteria for withdrawal (if applicable)

Allergic response to first SARS-CoV-2 vaccination.

e. Replacement of individual subjects after withdrawal

Subjects that previously had a SARS-CoV-2 infection will be excluded from the study as this compromises comparability between the groups. Initially, this will be determined by the inclusion questionnaire. However, asymptomatic infections with SARS-CoV-2 are a possibility, therefore serum obtained from study participants at the first time-point (pre-booster vaccination) will be screened for the presence of

nucleocapsid (N)-specific antibodies via ELISA. If a participant tests positive for the presence of N-specific antibodies, this subject will be excluded from the per protocol analysis. From data from the CoM-CoV study, it is expected that exclusion due to previous SARS-CoV-2 infection could be up to 25%. We have corrected for this in our sample size calculation. Although these participants are formally excluded from the downstream analyses, these seropositive participants will still be followed throughout the study.

f. Follow-up of subjects withdrawn from treatment

Participants who withdraw from the study will receive the same standard treatment as described in the then applicable guidelines. Already collected and distributed samples/ specimens and data will not be destroyed or deleted.
If the participants are excluded from the per protocol analysis, they will be followed and SoC will be guaranteed if guidelines advice this at that moment.

g. Premature termination of the study

If from the data it can be concluded that switching might compromise the immunogenicity of the participants, the study will be ended immediately and all necessary steps will be taken to safeguard the safety of the participants.

9. SAFETY REPORTING

h. Temporary halt for reasons of subject safety

In accordance to section 10, subsection 4, of the WMO, the sponsor will suspend the study if there is sufficient ground that continuation of the study will jeopardise subject health or safety. The sponsor will notify the accredited METC without undue delay of a temporary halt including the reason for such an action. The study will be suspended pending a further positive decision by the accredited METC. The investigator will take care that all subjects are kept informed.

i. AEs, SAEs and SUSARs

i. Adverse events (Aes)

Adverse events are defined as any undesirable experience occurring to a subject during the study, whether or not considered related to the trial procedure. All adverse events reported spontaneously by the subject or observed by the investigator or his staff will be recorded. However, given the setting of a large outbreak, the registration of Aes is not feasible and will not serve the safety of the participants. Therefore, Aes will not be reported.

ii. Adverse Reaction (AR)

An untoward and unintended response in a participant to an investigational medicinal product which is related to any dose administered to that participant.
The phrase "response to an investigational medicinal product" means that a causal relationship between a trial medication and an AE is at least a reasonable possibility, i.e. the relationship cannot be ruled out.
All cases judged by either the reporting medically qualified professional or the Sponsor as having a reasonable suspected causal relationship to the trial medication qualify as adverse reactions.

iii. Serious adverse events (SAEs)

A serious adverse event is any untoward medical occurrence or effect that

- results in death;
- is life threatening (at the time of the event);
- requires hospitalisation or prolongation of existing inpatients’ hospitalisation;
- results in persistent or significant disability or incapacity;
- is a congenital anomaly or birth defect; or

- any other important medical event that did not result in any of the outcomes listed above due to medical or surgical intervention but could have been based upon appropriate judgement by the investigator.

An elective hospital admission will not be considered as a serious adverse event.

The registration of SAEs will be limited to SAEs that result in life threatening events, requires hospitalization or death. SAEs will be reported only from the moment of inclusion until one day after the last blood taking after 365 days. The sponsor will report the SAEs through the web portal *ToetsingOnline* to the accredited METC that approved the protocol, within 7 days of first knowledge for SAEs that result in death or are life threatening followed by a period of maximum of 8 days to complete the initial preliminary report. All other SAEs will be reported within a period of maximum 15 days after the sponsor has first knowledge of the serious adverse events.

iv. Suspected unexpected serious adverse reactions (SUSARs)

Adverse reactions are all untoward and unintended responses to an investigational product related to any dose administered.
Unexpected adverse reactions are SUSARs if the following three conditions are met:
1. the event must be serious (see chapter 9.2.2);
2. there must be a certain degree of probability that the event is a harmful and an undesirable reaction to the medicinal product under investigation, regardless of the administered dose;
the adverse reaction must be unexpected, that is to say, the nature and severity of the adverse reaction are not in agreement with the product information as recorded in:
- Summary of Product Characteristics (SPC) for an authorized medicinal product;
- The sponsor will report expedited the following SUSARs through the web portal ToetsingOnline to the METC :
- SUSARs that have arisen in the clinical trial that was assessed by the METC;
- SUSARs that have arisen in other clinical trials of the same sponsor and with the same medicinal product, and that could have consequences for the safety of the subjects involved in the clinical trial that was assessed by the METC.
The remaining SUSARs are recorded in an overview list (line-listing) that will be submitted once every half year to the METC. This line-listing provides an overview of all SUSARs from the study medicine, accompanied by a brief report highlighting

the main points of concern.

The expedited reporting of SUSARs through the web portal Eudravigilance or ToetsingOnline is sufficient as notification to the competent authority.

The sponsor will report expedited all SUSARs to the competent authorities in other Member States, according to the requirements of the Member States.

The expedited reporting will occur not later than 15 days after the sponsor has first knowledge of the adverse reactions. For fatal or life threatening cases the term will be maximal 7 days for a preliminary report with another 8 days for completion of the report. Principal investigators of the participating centers in this multicenter study will report SUSARs to the coordinating investigator of this study and the coordinating investigator will be responsible for report the SUSARs as described above.

Assessment results outside of normal parameters as AEs and SAEs

Laboratory

Abnormal clinical findings from safety blood tests will be assessed by a medically qualified study member. Laboratory AEs will be assessed using specific toxicity grading scales adapted from the FDA Toxicity Grading Scale for Healthy Adult and Adolescent Volunteers Enrolled in Preventive Vaccine Clinical Trials **(APPENDIX A: Toxicity grading scale for lab AEs)**

Any abnormal test result deemed clinically significant may be repeated to ensure it is not a single occurrence, if deemed appropriate to do so in the medical opinion of the investigator.

If a repeated test remains clinically significant, the participant will be informed and appropriate medical care arranged as appropriate and with the permission of the volunteer.

Procedures for reporting Adverse Events

Solicited AEs

Participants will be asked to record local and systemic AE's for 7 days in their paper diary. Participants are asked to fill in these AE's in a digital questionnaire 7 days after boost.

Local solicited AEs; pain, redness, warmth, swelling

Systemic solicited AEs; joint pain, fatigue, fever, chills, headache, muscle pain, nausea

Unsolicited AEs

All local and systemic AEs occurring 28 days following boost (2nd vaccination) observed by the Investigator or reported by the participant. This will be actively asked in a digital questionnaire, and will be recorded in the study database.

Medically attended AEs

A medically attended AE, is defined as any adverse event for which the participant seeks medical attention either at hospital or from primary care. This explicitly excludes seeking medical attention solely for a SARS-CoV-2 test. Participants will be asked to record any medically attended AEs, either directly to the investigator or by filling in a questionnaire (every Study Visit). Medically attended AEs occurring up to 3 months post boost, will be directly solicited and reviewed at each study visit.

j. Annual safety report

In addition to the expedited reporting of SUSARs, the sponsor will submit, once a year throughout the clinical trial, a safety report to the accredited METC, competent authority, and competent authorities of the concerned Member States.

This safety report consists of:

- a list of all suspected (unexpected or expected) serious adverse reactions, along with an aggregated summary table of all reported serious adverse reactions, ordered by organ system, per study;
- a report concerning the safety of the subjects, consisting of a complete safety analysis and an evaluation of the balance between the efficacy and the harmfulness of the medicine under investigation.

k. Follow-up of adverse events

All SAEs will be followed until they have abated, or until a stable situation has been reached. Depending on the event, follow up may require additional tests or medical procedures as indicated, and/or referral to the general physician or a medical specialist. SAEs need to be reported till end of study within the Netherlands, as defined in the protocol.

l. [Data Safety Monitoring Board (DSMB) / Safety Committee]

If the subjects had not participated in this trial, they could have received the same vaccine via the national vaccination campaign with no additional safety surveillance. Furthermore,

vaccination of all participants will probably be completed within 8 weeks. Therefore, interim safety analysis is deemed not necessary and not feasible. Biweekly investigator meetings will be held until 4 weeks after the last patient has received the last vaccination and less frequently thereafter to discuss, among others, the different types of Aes.

10. STATISTICAL ANALYSIS

We will analyse the data in three steps. In the first step, the baseline characteristics are presented for the total sample (participants that are included on per protocol basis) and for each of the four arms separately. Continuous variables will be presented as mean ± standard deviation in case of a normal distribution or median (interquartile range) in case of non-normal distribution. Categorical variables will be presented as numbers (percentage). In addition, we will examine difference in baseline characteristics for the three comparisons between the arm that receives Janssen solely and the arms where Janssen is combined with a boost vaccination (i.e. Janssen, Moderna, or Pfizer). Baseline characteristics in each comparison will be compared with control groups using t-test or Mann-Whitney U-test (whichever appropriate based on the normality of the variable distribution) for continuous variables and Pearson Chi-square test for categorical variables. Normality of the distribution of the continuous variable will be assessed by the Shapiro-Wilk test.

For the primary outcome, measuring the immune response at day 28 after the boost/2nd vaccination (determination of antibodies by a quantitative IgG assay (LIAISON SARS-CoV-2 TrimericS IgG essay), we will compare the arm that only receives Janssen vaccination group with arm that receives Janssen/Janssen. Further, we will compare the Janssen/Janssen arm with the other two arms (i.e., Janssen/Pfizer, and Janssen/Moderna), separately. As the primary outcome is continuous, a log (base 10) transformation will be applied to the titer level, differences will be examined using an independent two-sample student T-test or Mann Whitney U test when the primary endpoint is normally distributed or not, respectively. Additionally, in the case that there are statistically significant differences in baseline characteristics between groups, we will use a linear regression to adjust for these imbalances.

In the next step, we will analyse our secondary outcomes. For our secondary outcome, we specified several endpoints: assess safety and reactogenicity of different boost COVID-19 vaccines schedules and characterization of immunogenicity of these schedules. These variables will be described and analysed as our baseline characteristics for all three comparisons between treatment arms, separately. The analysis will be done on a per-protocol basis. This analysis will include only the participants that were negative for the nucleocapsid (N)-specific antibody ELISA's test, that determines whether study participants previously experienced COVID-19 infection (as assumed to be COVID-19 negative before the first vaccination). An intention-to-treat analysis will be performed as secondary analysis. Taking into account a possible multiple-testing problem, we will use a p-value below 0.01 as a cut off to conclude statistical significance.

a. Primary study parameter(s)

The primary study parameter is the antibody response (as measures by LIAISON® SARS-CoV-2 TrimericS IgG assay) to SARS-CoV-2 28 days after 2nd vaccination. This endpoint is aligned with the other studies, such as the CoM-COV trial (8, 10).

b. Secondary study parameter(s)

1. To assess safety of homologous/heterologous prime-boost schedules of COVID-19 vaccines with Janssen/Moderna/Pfizer.
Outcome measures; (serious) adverse events during the study.

2.Reactogenicity and safety of homologous/heterologous prime-boost schedules of COVID-19 vaccines with Janssen/Moderna/Pfizer.
Outcome measures; solicited local & systemic reactions 7 days after immunization (prime as booster). Changes from baseline in laboratory safety measures (D0, D28, D180 and D365)

3.Further characterization of immunogenicity of homologous/heterologous prime-boost schedules of COVID-19 vaccines with Janssen/Moderna/Pfizer (D0, D28, D180 and D365).

4. Evaluation of immunogenicity, safety, and reactogenicity of COVID-19 vaccines in participants sero-positive for SARS-CoV-2 IgG at baseline.
Outcome measures; Immunogenicity, reactogenicity, and safety endpoints as outlined above. In addition to questionnaires, we will perform nucleocapsid (N)-specific antibody ELISA's to determine whether study participants previously experienced COVID-19.

c. Other study parameters

N/A

d. Interim analysis (if applicable)

N/A

11. ETHICAL CONSIDERATIONS

a. Regulation statement

The study will be performed in accordance with ethical principles that have their origin in the Declaration of Helsinki (64th version, October 2013) and are consistent with the International Conference on Harmonisation (ICH), Good Clinical Practice (GCP) guidelines, applicable regulatory requirements. The Investigator must also comply with all applicable privacy directives and regulations (e.g., EU Data protection Directive 95/46/EC). The principal investigator is responsible for the proper conduct of the study at the study site.

b. Recruitment and consent

Initially we want to focus on HCW vaccinated once with Janssen within hospitals. If insufficient participants are included, the study population will shift from HCW within hospitals to HCW in primary care (e.g. local pharmacies, dental practices & physiotherapists).

- HCWs within hospitals will be approached by e-mail and intranet.
- HCWs from primary care will be approached via e-mail or local contacts (using local GP practices / pharmacies).

Screening and Eligibility Assessment

Once participants express an interest in joining the trial, their e-mail address is connected to an online screening questionnaire for eligibility. This questionnaire will assess for exclusion criteria. If they pass this stage, they receive extra information (PIF).

ICH-GCP guidelines will be followed in informing the participant and obtaining consent. Before informed consent may be obtained, the participant will be given the time and opportunity to inquire about details of the trial and to decide whether or not to participate. All participants' questions about the trial will be answered before signing the informed consent form. Written informed consent of participants is required before enrolment in the trial and before any study related procedure will take place.

The content of the participant information letter, informed consent form and any other written information to be provided to participants will be in compliance with ICH-GCP, GDPR and other applicable regulations and will be approved by the ethics committee in advance of use. The participant information letter, informed consent form and any other written information to be provided to participants will be revised whenever important new

information becomes available that may be relevant to the participant's consent. Any substantially revised informed consent form and written information will be approved by the ethics committee in advance of use. The participant will be informed in a timely manner if new information becomes available that might be relevant to the participant's willingness to continue participation in the trial.

c. Objection by minors or incapacitated subjects (if applicable)

N/A

d. Benefits and risks assessment, group relatedness

1.The group of persons that will be approached for this study are all Health Care Workers vaccinated once with Janssen. The crucial importance of a second vaccination was recently demonstrated for the Indian variant B.1.617.2 in the UK. A single immunization with either Pfizer or AstraZeneca vaccine provided only 33% protection against symptomatic disease. A second immunization increased protection to 88% and 66% for the Pfizer and AstraZeneca vaccine respectively (15). Therefore it is important to examine boosting for people vaccinated once with Janssen.

2. If combination of two different vaccines will not lead to sufficient protection as seen in the titer of antibodies, this will be identified within 3 months after the second injection. This can be the case for both an individual or a group. If this appears, adequate measures will be taken to reassure the safety of the HCW. That means you will be called up for a 3rd vaccination. Which that will be determined in consultation with infectiologists/virologists and the applicable guidelines of RIVM. However, from preliminary oral report from CombiVacS it can be concluded that boost with a different vaccine enhances the immune response.

3. If it turns out during the study that a single-dose of Janssen is no longer part of standard care, but is advised to boost with Janssen or otherwise, we guarantee that we adhere to the guidelines that apply according to RIVM. This means that we guarantee you that you will receive a boost (2nd vaccination).

4. It is difficult to predict whether the occurrence of side effects (reactogenicity) will increase or decrease. Recently, an interim analysis of participants from the com-CoV study has shown that immunization with heterologous schedules of Astra Zeneca and Pfizer result in more frequent solicited systemic reactions such as fatigue, chills, feverishness and malaise than the homologous schedules for these vaccines. They reported the experienced systemic reactions in the first 48 hours after booster vaccination and were not associated with hospital admissions (11). In contrast, in a prospective study from Germany with the same vaccination strategy, they identified a comparable reactogenicity between heterologous (Astra

Zeneca/Pfizer) and homologous (Pfizer/Pfizer) booster vaccination after a 12-week dose interval (12). The heterologous combination from our study will have to reveal what the exact effect is on reactogenicity.
5. The occurrence of anaphylaxis might increase as the HCW are exposed to two different regimens. The incidence of anaphylaxis appears to be so low that this might not be of clinical significance. To mitigate the occurrence of anaphylaxis the HCW will be monitored after the vaccination for at least 15 minutes.
6. It is important to state that the vaccines for this study will NOT be retrieved from a stock reserved for patients or other persons. The vaccines will be supplied by the RIVM after approval by ZonMw and VWS.
7. As two different vaccines will be administered, it may be the case that through vaccinations with slightly different vaccines the immune-response may even be improved.
8. With the upcoming Variants of Concern (e.g. the British variant), it has been suggested that the present vaccines could be changed a bit to improve the vaccination against these variants. In that case it might even be a necessity to combine vaccines to cover several different VoC.
9. Each participant will receive a measurement of their immune response to SARS-CoV-2 and will know their immune titer approximately 6 weeks after the booster (2nd vaccination). This description indicates whether you are adequately protected or not. If someone is not properly protected, we will provide sufficient protection by administering an extra vaccine, see also under point 2.
10. As stated in the introduction of the study, due to shortages, logistic problems and reservations of vaccines for the 2nd booster, the velocity of vaccination is hampered all over the world. If this study concludes that combining different vaccines (heterologous vaccine regimen) gives a good immune response against SARS-CoV-2, this has a huge societal impact for the world.

Conclusion

Based on the considerations mentioned above and weighing both the risks and the benefits of this trial, we conclude that there is a positive risk-benefit analysis to proceed with this trial.

e. Compensation for injury

The sponsor/investigator has a liability insurance which is in accordance with article 7 of the WMO. The sponsor (also) has an insurance which is in accordance with the legal requirements in the Netherlands (Article 7 WMO). This insurance provides cover for damage to research subjects through injury or death caused by the study. The insurance applies to

the damage that becomes apparent during the study or within 4 years after the end of the study. The sponsor/investigator has a liability insurance which is in accordance with article 7 of the WMO. The sponsor (also) has an insurance which is in accordance with the legal requirements in the Netherlands (Article 7 WMO). This insurance provides cover for damage to research subjects through injury or death caused by the study. The insurance applies to the damage that becomes apparent during the study or within 4 years after the end of the study.

f. Incentives (if applicable)

N/A

12. ADMINISTRATIVE ASPECTS, MONITORING AND PUBLICATION

a. Handling and storage of data and documents

Data and documents will be controlled and processed conform the EU General Data Protection Regulation (GDPR) and the Dutch Act on Implementation of the General Data Protection Regulation. (in Dutch: Uitvoeringswet AVG, UAVG).

i. Confidentiality of the data

By signing this protocol, the investigator affirms to the Sponsor that information furnished to the investigator will be and such information will be divulged to the institutional review board, ethics review committee (IRB/ERC) or similar or expert committee; affiliated institution and employees, only under an appropriate understanding of confidentiality with such board or committee, affiliated institution and employees. Data generated by this trial will be considered confidential by the investigator, except to the extent that it is included in a publication as provided in the Publications section of this protocol.

ii. Confidentiality of subject records

Prior to enrollment in the study and prior to any study related procedure, the participant must personally sign and date the informed consent form.
Each participant will be given a unique sequential study subject number not based on the participant's initials or birthdate. The key to the participant study number is safeguarded by the local investigator.
Data will be collected using an eCRF (electronic case report form) designed for this study. According to ICH guidelines for Good Clinical Practice, the monitoring team must check the specific CRF entries against the source documents, as specified in the study specific monitoring plan. The Informed Consent Form will include a statement by which the participant allows the Sponsor's duly authorized personnel, the ethics review committee (IRB/ERC) or similar or expert committee, and the regulatory authorities to have direct access to original medical records which support the data on the CRFs (e.g. participant's medical file).
This personnel, bound by professional secrecy, must maintain the confidentiality of all personal identity or personal medical information, according to confidentiality and personal data protection rules and in compliance with all applicable privacy laws, rules, and regulations.

iii. Data Management

The investigator or qualified designee is responsible for recording and verifying the accuracy of subject data. By signing this protocol, the investigator acknowledges that his/her electronic signature is the legally binding equivalent of a written signature. By entering his/her electronic signature, the investigator confirms that all recorded data have been verified as accurate.

iv. Biological samples

Biological samples will only be stored for the purpose of additional research if the participant has given consent. Material that is not used for current translational research will be stored for up to 15 years after end of study. If no informed consent was obtained, samples will be destroyed after the participant has completed all protocol treatment and procedures. Storage of biological samples on site is subject to the site's guidelines.

b. Monitoring and Quality Assurance

On-site monitoring will take place according to the NFU (Nederlandse Federatie van Universitaire Medisch Centra)-guideline "Kwaliteitsborging van mensgebonden onderzoek 2019" by the appointed monitor. This study is classified as negligible risk because vaccination is standard of care. Monitoring will take place to assure the quality and validity of the research data. The monitor will perform source data verification on the research data by comparing the data entered into the CRF with the available source documentation and other available documents. Source documents are defined as the patient's hospital medical records, clinician notes, laboratory print outs, digital and hard copies of imaging, memos, electronic data etc.
The monitor will verify the following items: Patient flow (inclusion speed and dropout rate); Informed consent forms (presence, dates, signatures); Informed consent process, Trial Master File and Investigator Files (presence of all documents), in-/exclusion criteria (using source documents). After each control the monitor will send a written report to the sponsor (including a summary; quality assessment; summary of findings, deviations and shortcomings; possible solutions to warrant compliance with the protocol; final conclusion).

c. Amendments

Amendments are changes made to the research after a favourable opinion by the accredited METC has been given. All amendments will be notified to the METC that gave a favourable opinion.
All substantial amendments will be notified to the METC and to the competent authority.

Non-substantial amendments will not be notified to the accredited METC and the competent authority, but will be recorded and filed by the sponsor.

d. Annual progress report

The sponsor/investigator will submit a summary of the progress of the trial to the accredited METC once a year. Information will be provided on the date of inclusion of the first subject, numbers of subjects included and numbers of subjects that have completed the trial, serious adverse events/ serious adverse reactions, other problems, and amendments.

e. Temporary halt and (prematurely) end of study report

The investigator/sponsor will notify the accredited METC of the end of the study within a period of 8 weeks. The end of the study is defined as the last participant’s last visit.

The sponsor will notify the METC immediately of a temporary halt of the study, including the reason of such an action.

In case the study is ended prematurely, the sponsor will notify the accredited METC within 15 days, including the reasons for the premature termination.

Within one year after the end of the study, the investigator/sponsor will submit a final study report with the results of the study, including any publications/abstracts of the study, to the accredited METC.

f. Public disclosure and publication policy

Trial results will be submitted for publication in a peer reviewed scientific journal regardless of the outcome of the trial. Results may not be published if the trial was terminated prematurely and/ or did not yield sufficient data for a publication.

13. STRUCTURED RISK - BENEFIT ANALYSIS

1.The group of persons that will be approached for this study are all Health Care Workers vaccinated once with Janssen. The crucial importance of a second vaccination was recently demonstrated for the Indian variant B.1.617.2 in the UK. A single immunization with either Pfizer or AstraZeneca vaccine provided only 33% protection against symptomatic disease. A second immunization increased protection to 88% and 66% for the Pfizer and AstraZeneca vaccine respectively (15). Therefore It is important to examine boosting for people vaccinated once with Janssen.

2. If combination of two different vaccines will not lead to sufficient protection as seen in the titer of antibodies, this will be identified within 3 months after the second injection. This can be the case for both an individual or a group. If this appears, adequate measures will be taken to reassure the safety of the HCW. That means you will be called up for a 3rd vaccination. Which that will be determined in consultation with infectiologists/virologists and the applicable guidelines of RIVM.

3. If it turns out during the study that a single-dose of Janssen is no longer part of standard care, but is advised to boost with Janssen or otherwise, we guarantee that we adhere to the guidelines that apply according to RIVM. This means that we guarantee you that you will receive a boost (2nd vaccination).

4. It is difficult to predict whether the occurrence of side effects (reactogenicity) will increase or decrease. Recently, an interim analysis of participants from the com-CoV study has shown that immunization with heterologous schedules of Astra Zeneca and Pfizer result in more frequent solicited systemic reactions such as fatigue, chills, feverishness and malaise than the homologous schedules for these vaccines. They reported the experienced systemic reactions in the first 48 hours after booster vaccination and were not associated with hospital admissions (11). In contrast, in a prospective study from Germany with the same vaccination strategy, they identified a comparable reactogenicity between heterologous (Astra Zeneca/Pfizer) and homologous (Pfizer/Pfizer) booster vaccination after a 12-week dose interval (12). The heterologous combination from our study will have to reveal what the exact effect is on reactogenicity.

5. The occurrence of anaphylaxis might increase as the HCW are exposed to two different regimens. The incidence of anaphylaxis appears to be so low that this might not be of clinical significance. To mitigate the occurrence of anaphylaxis the HCW will be monitored after the vaccination for at least 15 minutes.

6. It is important to state that the vaccines for this study will NOT be retrieved from a stock reserved for patients or other persons. The vaccines will be supplied by the RIVM after approval by ZonMw and VWS.

7. As two different vaccines will be administered, it may be the case that through vaccinations with slightly different vaccines the immune-response may even be improved.
8. With the upcoming Variants of Concern (e.g. the British variant), it has been suggested that the present vaccines could be changed a bit to improve the vaccination against these variants. In that case it might even be a necessity to combine vaccines to cover several different VoC.
9. Each participant will receive a measurement of their immune response to SARS-CoV-2 and will know their immune titer approximately 6 weeks after the booster (2nd vaccination). This description indicates whether you are adequately protected or not. If someone is not properly protected, we will provide sufficient protection by administering an extra vaccine, see also under point 2.
10. As stated in the introduction of the study, due to shortages, logistic problems and reservations of vaccines for the 2nd booster, the velocity of vaccination is hampered all over the world. If this study concludes that combining different vaccines (heterologous vaccine regimen) gives a good immune response against SARS-CoV-2, this has a huge societal impact for the world.

Conclusion

Based on the considerations mentioned above and weighing both the risks and the benefits of this trial, we conclude that there is a positive risk-benefit analysis to proceed with this trial.

14. REFERENCES

1. World Health Organization. Disease Outbreak News: Pneumonia of unknown cause – China. Available at: https://www.who.int/csr/don/05-january-2020-pneumonia-of-unkown-causechina/en/.
2. Voysey M, Costa Clemens SA, Madhi SA, Weckx LY, Folegatti PM, Aley PK, et al. Safety and efficacy of the ChAdOx1 nCoV-19 vaccine (AZD1222) against SARS-CoV-2: an interim analysis of four randomised controlled trials in Brazil, South Africa, and the UK. Lancet. 2021(397): 99–111.
3. Baden LR, El Sahly HM, Essink B, Kotloff K, Frey S, Novak R, et al. Efficacy and Safety of the mRNA-1273 SARS-CoV-2 Vaccine. N Engl J Med. 2021;384(5):403-16.
4. Polack FP, Thomas SJ, Kitchin N, Absalon J, Gurtman A, Lockhart S, et al. Safety and Efficacy of the BNT162b2 mRNA Covid-19 Vaccine. N Engl J Med. 2020;383(27):2603-15.
5. H. L. Could mixing COVID vaccines boost immune response? Nature. 2021(590):375–6.

6. He Q, Mao Q, An C, Zhang J, Gao F, Bian L, et al. Heterologous prime-boost: breaking the protective immune response bottleneck of COVID-19 vaccine candidates. Emerg Microbes Infect. 2021;10(1):629-37.
7. Spencer AJ, McKay PF, Belij-Rammerstorfer S, Ulaszewska M, Bissett CD, Hu K, et al. Heterologous vaccination regimens with self-amplifying RNA and adenoviral COVID vaccines induce robust immune responses in mice. Nat Commun. 2021;12(1):2893.
8. Snape MD, Heath P, Faust S, Finn A, Green C, Lazarus R, et al. Protocol Com-COV: A single-blind, randomised, phase II UK multi-centre study to determine reactogenicity and immunogenicity of heterologous prime/boost COVID-19 vaccine schedules. 2021.
9. Protocol CombiVacS: A Phase 2, Comparative, Randomised, Adaptive Trial to Evaluate the safety and immunogenicity of one dose of COMIRNATY® in subjects that had received one dose of VAXZEVRIA. 2021.
10. Snape MD, Ramasamy M, Heath P, Green C, Turner D, Collins A, et al. Protocol Com-COV2: A single-blind, randomised, phase II UK multi-centre study to determine reactogenicity and immunogenicity of heterologous prime/boost COVID-19 vaccine schedules – Stage 2. 2021.
11. Shaw RH, Stuart A, Greenland M, Liu X, Nguyen-Van-Tam JS, Snape MD. Heterologous prime-boost COVID-19 vaccination: initial reactogenicity data. Lancet. 2021.
12. Hillus D, Tober-Lau P, Hastor H, Helbig ET, Lippert LJ, Thibeault C, et al. Reactogenicity of homologous and heterologous prime-boost immunisation with BNT162b2 and ChAdOx1-nCoV19: a prospective cohort study. medRxiv. 2021.
13. Sadoff J, Gray G, Vandebosch A, Cardenas V, Shukarev G, Grinsztejn B, et al. Safety and Efficacy of Single-Dose Ad26.COV2.S Vaccine against Covid-19. N Engl J Med. 2021.
14. Ensemble 2 protocol: A Randomized, Double-blind, Placebo-controlled Phase 3 Study to Assess the Efficacy and
Safety of Ad26.COV2.S for the Prevention of SARS-CoV-2-mediated COVID-19 in Adults Aged 18 Years and Older. 2020.
15. Public Health England - Vaccines highly effective against B.1.617.2 variant after 2 doses (https://www.gov.uk/government/news/vaccines-highly-effective-against-b-1-617-2-variant-after-2-doses).
16. Voysey M, Costa Clemens SA, madhi SA, Weckx LY, Folegatti PM, Aley PK, et al. Single dose administration, and the influence of the timing of the booster dose on immunogenicity and efficacy of ChAdOx1 nCoV-19 (AZD1222) vaccine. Preprints. 2021.
17. Wise J. Covid-19: New data on Oxford AstraZeneca vaccine backs 12 week dosing interval. BMJ. 2021;372:n326.

18. van Kampen JJA, van de Vijver D, Fraaij PLA, Haagmans BL, Lamers MM, Okba N, et al. Duration and key determinants of infectious virus shedding in hospitalized patients with coronavirus disease-2019 (COVID-19). Nat Commun. 2021;12(1):267.
19. den Hartog G, Schepp RM, Kuijer M, GeurtsvanKessel C, van Beek J, Rots N, et al. SARS-CoV-2-Specific Antibody Detection for Seroepidemiology: A Multiplex Analysis Approach Accounting for Accurate Seroprevalence. J Infect Dis. 2020;222(9):1452-61.
20. GeurtsvanKessel CH, Okba NMA, Igloi Z, Bogers S, Embregts CWE, Laksono BM, et al. An evaluation of COVID-19 serological assays informs future diagnostics and exposure assessment. Nat Commun. 2020;11(1):3436.
21. Okba NMA, Muller MA, Li W, Wang C, GeurtsvanKessel CH, Corman VM, et al. Severe Acute Respiratory Syndrome Coronavirus 2-Specific Antibody Responses in Coronavirus Disease Patients. Emerg Infect Dis. 2020;26(7):1478-88.
22. Geers D, Shamier MC, Bogers S, Den Hartog G, Gommers L, Nieuwkoop NN, et al. SARS-CoV-2 variants of concern partially escape humoral but not T-cell responses in COVID-19 convalescent donors and vaccinees. Science Immunology. 2021.
23. Weiskopf D, Schmitz KS, Raadsen MP, Grifoni A, Okba NMA, Endeman H, et al. Phenotype and kinetics of SARS-CoV-2–specific T cells in COVID-19 patients with acute respiratory distress syndrome. Science Immunology. 2020;5.

Appendix A: Toxicity grading scale for lab AEs

**Haematology**		**Units**	**Lab range**	**Grade 1**	**Grade 2**	**Grade 3**	**Grade 4**
Haemoglobin	Male	mmol/L	8.1-10.5	7.1-7.8	6.2-7.0	5.3-6.1	<5.3
Haemoglobin	Female	mmol/L	7.5-9.3	6.5-7.0	5.6-6.4	5.0-5.5	<5.0
Leukocytes	Elevated	x 109/L	11.0	11.50-15.00	15.01-20.00	20.01-25.00	>25.00

Leukocytes	Low	x 109/L	4.0	2.50-3.50	1.50-2.49	1.00-1.49	<1.00
Platelets	Low	x 109/L	150-400	125-140	100-124	25-99	<25
Neutrophils	Low	x 109/L	2.00-7.00	1.50-1.99	1.00-1.49	0.50-0.99	<0.50
Lymphocytes	Low	x 109/L	1.00-4.00	0.75-0.99	0.50-0.74	0.25-0.49	<0.25
Eosinophils	Elevated	x 109/L	0.02-0.50	0.65-1.50	1.51-5.00	>5.00	Hypereosinophilia

**Biochemistry**		**Units**	**Lab range**	**Grade 1**	**Grade 2**	**Grade 3**	**Grade 4**
**Sodium**	**Elevated**	**mmol/L**	**145**	146-147	148-149	150-155	>155
**Sodium**	**Low**	**mmol/L**	**135**	132-134	130-131	125-129	<125
**Potassium**	**Elevated**	**mmol/L**	**5.0**	5.1-5.2	5.3-5.4	5.5-6.5	>6.5
**Potassium**	**Low**	**mmol/L**	**3.5**	3.2-3.3	3.1	2.5-3.0	<2.5
**Urea**	**Elevated**	**mmol/L**	**2.5-7.4**	8.2-9.3	9.4-11.0	>11.0	Requires dialysis
**Creatinine**	**Elevated**	**µmol/L**	**49-104**	1.1-1.5xULN<br>114-156	>1.5-3.0xULN<br>157-312	>3.0xULN<br>>312	Requires dialysis
**Bilirubin**	**Elevated***	**µmol/L**	**0-21**	1.1-1.5xULN<br>23-32	>1.5-2xULN<br>33-42	>2-3xULN<br>43-63	>3xULN<br>>63
**Bilirubin**	**Elevated***	**µmol/L**	**0-21**	1.1-1.25xULN<br>23-26	>1.25-1.5xULN<br>27-32	>1.5-1.75xULN<br>33-37	>1.75xULN<br>>37
**ALT**	**Elevated**	**IU/L**	**10-45**	1.1-2.5xULN<br>49-112	>2.5-5xULN<br>113-225	>5-10xULN<br>226-450	>10xULN<br>>450

**Alkaline Phosphatase**	**Elevated**	**IU/L**	**30-130**	1.1-2xULN 143-260	>2-3xULN 261-390	>3-10xULN 391-1300	>10xULN >1300
**Albumin**	**Low**	**g/L**	**32-50**	28-31	25-27	<25	-
**CRP**	**Elevated**	**Mg/L**	**0-10**	11-30	31-100	101-200	>200

***Normal ranges may vary between sites and gradings may be adapted between sites**
